# Technical Note: A new TLD‐phantom measurement system for determining dose distribution levels in the right and left breast from spiral CT chest imaging

**DOI:** 10.1120/jacmp.v3i4.2558

**Published:** 2002-09-01

**Authors:** Jeffery L. Hall, Jorge L. Navarrete, Edgar Surprenant, Jack Sklansky, Jack I. Eisenman

**Affiliations:** ^1^ Department of Radiology Martin Luther King/Drew Medical Center 12021 South Wilmington Boulevard Los Angeles California 90059

**Keywords:** CT dosimetry, computed tomography, breast, radiation dose, helical CT

## Abstract

Two specially designed plastic/aluminum phantoms positioned thermoluminescent dosimeters (TLDs) at the right and left breast location of an anthrophomorophic chest torso. Imaging was performed on a spiral CT for a volume of the chest phantom through the breast area for a noncontiguous (pitch 1.5) helical chest scan. Conventional pencil beam ionization chamber measurements were made at the same operating parameters. The doses ranged from approximately 1 to 3 cGy. For both breast phantoms, the doses were highest for the medial inner quadrants near the mediastinum. The doses were lowest for the outer quadrants (lateral aspects) of both breasts.

PACS number(s): 87.66.–a, 87.52.–g

## INTRODUCTION

Helical or spiral computed tomography has become a regular, high use tool for patient screening and diagnosis since this technique was introduced into clinical practice in 1989.^1^ ^,^ ^2^ Patient doses from CT examinations are above most of the other types of diagnostic x‐ray examinations.^3^ ^,^ ^4^ Previous studies have shown, for example in the United Kingdom, CT examinations that have been estimated to account for about 2.4% of all radiographic examinations, but account for about 20% of the annual collective dose from medical x‐rays.^5^ The increased speed of helical scanning allows the entire thorax to be scanned within a single breathold.^2^ ^–^ ^5^ Fast volumetric scanning allows acquisition of overlapping millimeter section images, and high definition three‐dimensional imaging of organs.

The radio sensitivity of the breast is high in comparison to other organs in the body. The choice of image acquisition factors (i.e., kVp and mAs) affects the dose to the breasts. The choice of these protocol imaging factors is determined by a resulting image quality sufficient for the required optimum diagnostic information. It is therefore important to investigate how much radiation exposure each quadrant of each breast receives in adults undergoing radiographic examinations with helical CT.

## METHODS

**Figure 1:**
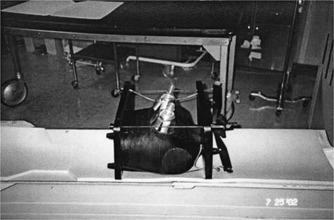
TLD‐phantom measurement system for determining dose distribution levels in the right and left breast from spiral CT chest imaging.

A third‐generation helical CT scanner (Marconi PQ 6000, Cleveland, OH) was used to perform helical scans. The two TLD attachment devices were constructed to accurately position TLDs around the right and left breast of a tissue equivalent phantom. The setup is shown in Fig. 1. Each breast TLD holding device was constructed of eight radially aligned aluminum strips 60 mm long, 25 mm wide, and 0.1 mm thick. The strips were attached to a central circular aluminum “hub” measuring 60 mm in diameter. The aluminum strips were held in contact with the breast by a plastic dome 115 mm high and 105 mm diameter at the base. The plastic material was 0.25 mm thickness hard plastic. The aluminum strips could be rotated independently of the outer plastic dome. One TLD capsule was attached to each of the 16 aluminum strips and an additional TLD was attached to the center or breast nipple area. The natural LiF TLDs were supplied and read by an independent company (Radiation Detection Company, Gilroy, CA). Both right and left breast phantoms were part of an anthrophomorophic upper body phantom. The aluminum strips were aligned with all four quadrants of each breast. CT images at 120 kVp of the phantom appear to have attenuation close to CT images of normal breast tissue. The thin (0.1 mm) aluminum strips did not alter the effective atomic number $\left(Z_{\text{eff}}\right)$ or electron density of the tissue‐equivalent phantom. A calculation^6^ of the effective atomic number for the phantom was 7.45 (water=7.42). Multidirectional, 120 kVp x‐ray attenuation (scatter and absorption) are functions of material density, atomic number, x‐ray energy, and thickness of material. The body phantom can simulate different breast sizes and breast densities for dose determination.

## RESULTS

**Figure 2:**
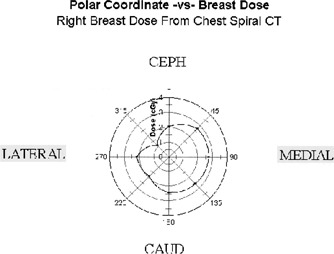
Dose distribution from spiral CT chest imaging. Eight different points surrounding the breast. Polar coordinate system, 80 mm radius centered at the nipple. Dose in cGy is shown as a function of eight angles, with computed linear interpolation.

**Figure 3:**
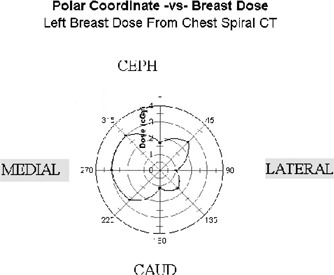
Dose distribution from spiral CT chest imaging. Eight different points surrounding the breast. Polar coordinate system, 80 mm radius centered at the nipple. Dose in cGy is shown as a function of eight angles, with computed linear interpolation.

Figures 2 and 3 show the dose at each breast using a polar coordinate system. The eight angles represent orientations of the aluminum strip. The dose in cGy for each angle is shown. The control settings for the CT scanner were 120 kVp, 200 mA, and a 1.5 pitch with a slice thickness of 8 mm. The resulting doses ranged from 1 to 3 cGy. This is about ten times the dose from one screen/film mammogram.^7^ As part of our verification, these results were compared to the results from a conventional pencil ion chamber measurement in a standard 30 centimeter diameter acrylic abdomen cylindrical phantom and with abdomen cylindrical phantom dose values reported in the literature^8^ ^,^ ^9^ for spiral CT systems. The doses were within the range of CTDI values reported. The center value was 0.86 cGy and average surface dose was 1.64 cGy (range 1.17 to 1.87 cGy). Measurements with a 20 cm diameter acrylic phantom for the same control settings resulted in a center value of 2.39 cGy, and average surface dose was 2.60 cGy (range 2.52 to 3.01 cGy).

## DISCUSSION

Patient dose in spiral CT is based on predetermined technique factors and protocols developed by CT manufacturers for adults. Current CT scanners do not adjust the radiation dose depending on how much the body attenuates during CT chest imaging. Unlike with CT, the patient dose in conventional film/screen mammography does depend upon how much the breast absorbs during the imaging procedure. Larger, denser breasts receive more radiation dose than normal breasts to insure the optimum range of film density on the mammogram. The more radiation used beyond what is optimum for the film, the worse the mammogram. With spiral CT chest imaging at our facility, the image acquisition factors are the same for all adult men and women without regard to breast size.

The expected benefit must be considered when reviewing the radiation dose to the breast during a chest CT examination. The amount of radiation that is given should be balanced against the image quality needed for a satisfactory diagnosis.

## CONCLUSION

The observed radiation dose to the breast with spiral chest CT ranged from 1 to 3 cGy. This is about ten times the dose from one screen/film mammogram. The medial portion of each breast received the highest dose.

The procedure and TLD phantoms in this study can be used for determining reference dose levels for future 3D breast volume CT imaging examinations. New protocols may vary with patient breast size and density. Radiation dose measurement of patient breasts at multiple locations with spiral CT is a step toward ensuring that no unnecessary radiation is used, and that the corresponding patient doses are minimized. Spiral CT chest imaging has greatly improved the diagnosis of many diseases and trauma. It is important to always be careful how we use it.

## References

[1] C. R. Crawford and K. F. King , “Computed tomography scanning with simultaneous patient translation,” Med. Phys. 17, 967–982 (1990). 2280740 10.1118/1.596464

[2] W. A. Kalender , W. Seissler , E. Klotz , and P. Vock , “Spiral volumetric CT with single‐breath‐hold technique, continuous transport, and continuous scanner rotation,” Radiology 176, 181–183 (1990). 2353088 10.1148/radiology.176.1.2353088

[3] National Council on Radiation Protection and Measurements (NCRP) , Report No. 100, “Exposure of the U.S. population from diagnostic medical radiation,” NCRP, Bethesda, MD (1989).

[4] United Nations Scientific Committee on the Effects of Atomic Radiation (UNSCEAR) , 1993 Report to the General Assembly, “Sources and effects of ionizing radiation,” United Nations, New York (1993).

[5] P. C. Shrimpton , D. Hart , M. C. Hillier , B. F. Wall , and K. Faulkner , “Survey of CT practice in the UK.1. Aspects of examination frequency and quality assurance,” National Radiological Protection Board (NRPB) Report No. R248, NRPB, Chilton, England, Her Majesty's Stationary Office (1991).

[6] P. Sprawls , The Principles of Diagnostic Radiology (University Park Press, Baltimore, 1977), pp. 30–33.

[7] K. L. Prado , J. T. Rakowski , F. Barragan , and K. N. Vanek , “Breast Radiation Dose in Film/Screen Mammography,” Health Phys. 55, 81–83 (1988). 3391781

[8] C. H. McCollough and F. E. Zink , “Performance evaluation of a multi‐slice CT system,” Med. Phys. 26, 2223–2230 (1999). 10587202 10.1118/1.598777

[9] J. T. Bushberg , The Essential Physics of Medical Imaging (Williams and Wilkins, Baltimore, 1994), p. 270, Table 10.3.

